# Duo photoprotective effect via silica-coated zinc oxide nanoparticles and Vitamin C nanovesicles composites

**DOI:** 10.1007/s11095-024-03733-y

**Published:** 2024-07-12

**Authors:** Soha M. Kandil, Heba M. Diab, Amal M. Mahfoz, Ahmed Elhawatky, Ebtsam M. Abdou

**Affiliations:** 1 Department of Pharmaceutics and Industrial Pharmacy, Faculty of Pharmacy, Modern University of Technology and Information (MTI), Cairo, Egypt; 2 Department of Dermatology, Venereology and Andrology, Faculty of Medicine, Ain Shams University, Cairo, Egypt; 3 Department of Pharmacology and Toxicology, Faculty of Pharmacy, Modern University of Technology and Information (MTI), Cairo, Egypt; 4 Department of Dermatology, Venereology and Andrology, National Research Centre, Cairo, Egypt; 5 Department of Pharmaceutics, Egyptian Drug Authority (EDA), former; National Organization of Drug Control and Research (NODCAR), Cairo, Egypt

**Keywords:** antera 3D® camera, ethosomes, magnesium ascorbyl phosphate (MAP), NF-κB, niosomes, photodamage, silica, UV irradiation, Zno

## Abstract

**Objective:**

Zinc Oxide nanoparticles (ZnO NPs) are used widely in nowadays personal care products, especially sunscreens, as a protector against UV irradiation. Yet, they have some reports of potential toxicity. Silica is widely used to cage ZnO NPs to reduce their potential toxicity. Vitamin C derivative, Magnesium Ascorpyl Phosphate (MAP), is a potent antioxidant that can efficiently protect human skin from harmful impacts of UV irradiation and oxidative stress. The combination of silica coated ZnO NPs and MAP nanovesicles could have potential synergistic protective effect against skin photodamage.

**Methods:**

Silica coated ZnO NPs and MAP nanovesicles (ethosomes and niosomes) were synthesized, formulated, and evaluated as topical gels. These gel formulations were evaluated in mice for their photoprotective effect against UV irradiation through histopathology and immuno-histochemistry study. Split-face clinical study was conducted to compare the effect of application of silica coated ZnO NPs either alone or combined with MAP nanovesicles. Their photoprotective action was evaluated, using Antera 3D® camera, for melanin level, roughness index and wrinkles depth.

**Results:**

Silica coated ZnO NPs when combined with MAP nanovesicles protected mice skin from UV irradiation and decreased the expression of the proinflammatory cytokines, NF-κB. Clinically, silica coated ZnO NPs, alone or combined with MAP nanovesicles, could have significant effect to decrease melanin level, roughness index and wrinkles depth with higher effect for the combination.

**Conclusion:**

A composite of silica coated ZnO NPs and MAP nanovesicles could be a promising cosmetic formulation for skin protection against photodamage signs such as hyperpigmentation, roughness, and wrinkles.

**Graphical Abstract:**

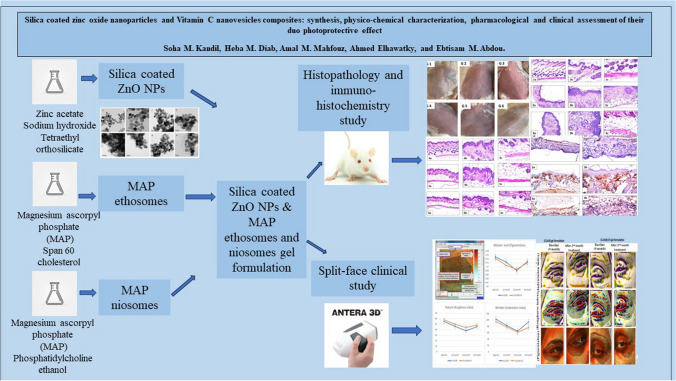

**Supplementary Information:**

The online version contains supplementary material available at 10.1007/s11095-024-03733-y.

## Introduction

Nanotechnology, including nano-scaled particles, is one of the foremost areas in the field of drug delivery when compared to bulk materials. Zinc oxide nanoparticles (ZnO NPs), being inorganic metal oxide nanoparticles, are widely used in variety of products and there is a progressing need for them along with other metal oxides [1, 2].

Currently, ZnO NPs are being largely used as a protector against UV irradiation in various skin care products, especially sunscreen creams and lotions, because of their capacity of blocking ultraviolet radiation [3]. Unfortunately, an increased number of nano-sized particles could potentially cause elevated risks to human beings as they, with their nano-size, can enter the biological systems through different exposure methods. Studies about safety and toxicity of inorganic nanoparticles are being reported. However, toxicological risks are still limited for most inorganic nanoparticles especially ZnO NPs [4, 5]. One of the suggested reasons for nanoparticles harmfulness is produced from the metal-based nanoparticles resulting in oxidative stress. Furthermore, after strong light absorption by the photocatalyst, NPs can produce free electrons and holes, the matter which produces reactive oxygen species by strong oxidation [6–9] making NPs have potential toxicity to humans and environments [10].

Many strategies are currently used to reduce this potential toxicity. One broadcasting strategy is based on coating the prepared ZnO NPs with inorganic supports, in particular, silica. Since the uncontrolled dispersion of NPs in the environment could be reduced by presence of suitable support, using silica coat could be beneficial [11–15]. Thus, silica is widely used to cage ZnO NPs to reduce the production of free radicals and reduce their potential toxicity [16, 17].

Another benefit of surface modifying of ZnO NPs with silica is to limit their coalescence and prevent aggregation growth [18]. This tendency to coalesce and agglomerate is still present despite being ZnO NPs prepared by many different methods [19].

Skin photodamage is known to be skin tissues specific damage that can be produced either by single or repeated exposure to ultraviolet (UV) irradiation. Erythema, fine and coarse wrinkles, edema, telangiectasia, dyspigmentation, sallowness, and roughness are the most predominant characteristics of skin photodamage [20, 21]. Skin photodamage represents an important concern of cosmetic, psychological, and physical issue for the patient because of the caused poor appearance and the risk of malignant tumors progression. Nowadays, there are progressing evidence reports about the involvement of UV-induced reactive oxygen species (ROS) generation and nuclear factor (NF-κB) activation in the pathogenesis flow of skin photodamage [22, 23]. NF-κB has a vital role in the photo-oxidative damage reduction and regulation of cell protection against UV irradiation. Moderate ROS from sun irradiation could induce NF-κB activation which is reported to regulate the expression of different inflammatory genes as inducible nitric oxide synthase (iNOS) [23]. As a result, reduction of the antioxidant defense system occurs with exacerbation of oxidative stress. These lead to destruction of the cutaneous cells and finally resulted in photoaging, photodamage, and photocarcinogenesis. As reported, NF-κB is one link among different molecular links between inflammation and cancer [24, 25].

Our previous studies showed that Magnesium ascorbyl Phosphate MAP, a vitamin C derivative, as a potent antioxidant when formulated into different nanoparticles, such as aspasomes, niosomes and ethosomes, could efficiently protect skin from the harmful impact of ROS and other photodamage signs [26, 27].

So, the aim of the current study was to investigate the photoprotective effect of prepared and evaluated silica coated ZnO NPs and MAP nanovesicles composites.

## Methods

### Materials

Zinc acetate dihydrate was purchased from Loba Chimie, India. Tetraethyl orthosilicate (TEOS); was obtained from Sigma Aldrich Company. Potassium hydroxide, Methanol HPLC grade, Absolute Ethanol ACS, REAG, isopropanol 99.9% and Ammonia 28% were obtained from Merck Millipore, Germany. Magnesium ascorbyl phosphate (MAP); was purchased from SOST, China. Soybean lecithin Phosphatidylcholine (PC), ≥ 94% PC was purchased from Alfa Aesar. Carbopol 934 was obtained from ADWIC Co., Cairo, Egypt. Methylene blue (MB), pharmaceutical grade (SRL, India). All used ingredients were of analytical grade.

### Preparation of Zinc Oxide nanoparticles (ZnO NPs)

ZnO NPs were prepared using a previously reported alkali co-precipitation method [28, 29]. Stoichiometric amounts of Zinc acetate and Sodium hydroxide were dissolved separately in 100 mL water while stirring at room temperature. After dissolving, Zinc acetate solution was heated up to 85 ºC for 30 min. At this stage, NaOH was added dropwise to the mixture. A white precipitate was formed, and the mixture was stirred for 2 h at 80 °C to ensure complete crystallization and growth of the nanoparticles. After centrifugation at 5,000 rpm, NPs were filtered, washed then dried at 120°C for 12 h. The particles were then thermally treated at 400 ºC for three hours.

### Synthesis of silica coated ZnO NPs (Si-ZnO NPs)

Silica-coated ZnO NPs were synthesized using previously reported sol–gel process [30, 31] with slight modification. In brief, ZnO nano powder (1g) was dispersed in alcoholic solution of ethanol and isopropanol (2:1 v/v) mixture. One mL of water and 28% aqueous NH_3_ were added into the mixture. This solution was sonicated 30 min. Tetraethyl orthosilicate (TEOS) was added drop by drop to the mixture. The reaction proceeded at room temperature for eight hours while stirring was continued. Si-ZnO NPs were collected by centrifugation at 10,000 rpm and washed three times with ethanol, then dried. Three formulations were prepared with three different TEOS concentrations relative to ZnO NPs and labelled as follow: ZnO NPs: TEOS in 1:4 ratio (Si-ZnO4), in 1:6 ratio (Si-ZnO6), 1:8 ratio (Si-ZnO8) in addition to the un-coated nanoparticles (ZnO NPs).

### Physico-chemical characterization of the prepared ZnO and Si-ZnO NPs

#### UV–vis spectra (UV–Vis) and Photoluminescence (PL)

The absorption spectra of the prepared nanoparticles under a UV–vis spectra (UV–Vis) was recorded using UV–Vis spectrophotometer, in a range of 200–2000 nm, equipped with a 150 mm integration sphere. Photoluminescence (PL) measurements were conducted at room temperature with laser wavelength of 365 nm, with line width of 15 ps, and a repetition rate of 10 MHz. The PL signal was recorded via Lumina Fluorescence Spectrometer- Thermo-Scientific.

#### Crystal Structure: X- ray diffraction

The crystal structure of the prepared NPs was performed using Empyrean Malvern analytical, Netherland X- ray diffraction. Samples were heated into non-hermetically aluminum pans at the rate of 20 ºC/minute with 2 theta (5.0º—100º), with step size 2Theta: 0.04 and at (Kα) = 1.54060º.

#### Morphological characterization using Transmission *Electron* Microscope (TEM)

Transmission electron microscopy morphological characterization of the prepared ZnO NPs and Si-ZnO NPs was conducted using high resolution transmission electron microscope (JEOL JEM-2100) to determine their shape and size at an accelerating voltage of 200 kV. Samples for TEM were prepared using droplets of the colloid suspension in a respective solvent on a Formvar carbon-coated, 300-mesh copper grid (Ted Pella) and allowing them to evaporate in air at ambient conditions.

#### Particles size and zeta potential measurements

Particle size (PS) analysis and zeta potential (ZP) measurement of the prepared samples were carried out using Zetasizer (Nano ZS90, Malvern Instrument, Worcestershire, UK). For accurate size measurement through dynamic light scattering (DLS), He–Ne laser (633 nm) was used as a light source. The scattered light was collected by using an avalanche photodiode detector (APD). The Z-Ave value; was described as the mean diameter of the nanoparticles [32]. ZP determination of the prepared samples were conducted using standard operation conditions with samples were appropriately diluted with de-ionized water. The results were expressed as the mean values (*n* = 3) ± SD.

#### Elemental analysis with energy dispersive X-ray spectroscopy (EDX)

The elemental composition of dried nanoparticles of both ZnO NPs and Si-ZnO8 NPs was determined using EDX analysis at an accelerating voltage of 100 kV.

#### Photocatalytic activity assessment and evaluation of reactive oxygen species (ROS) reduction

The photocatalytic activity of the prepared ZnO NPs and Si-ZnO8 NPs was evaluated through degradation of a methylene blue (MB) solution against UV irradiation as described previously [33, 34] with slight modification. A UVA lamp (F 20W/T12/BL368; SYLVANIA; GERMANY) with a peak irradiance of 368 nm was used as UV source for UV irradiation. MB dye solution was prepared at concentration of 0.004 g/L and 80 mL of this solution were irradiated with UV irradiation at 15 cm distance in presence of either 100 mg ZnO NPs or 240 mg Si-ZnO8 NPs (which contains nearly equivalent to 100 mg ZnO NPs). MB solution was used as a control. Before irradiation, the mixture of MB and NPs was magnetically stirred in the dark for 60 min to establish the adsorption–desorption equilibrium. During irradiation, 2 mL MB was drawn at fixed time interval each 15 min, filtered and absorbance was measured at 665 nm using UV–Vis spectrophotometer. The UV shielding performance (I) of the samples was calculated using the following equation:

$$\mathrm I={\mathrm A}_{\mathrm t/{\mathrm A}_0\times100.}$$

Where A_t_ represents the absorbance of the MB at time (t) and A_0_ represents the initial absorbance. Results were plotted against time.

### Preparation and Physico-chemical characterization of Magnesium-ascorpyl phosphate (MAP) nanovesicles (niosomes and ethosomes)

Magnesium ascorpyl phosphate (MAP) niosomes and ethosomes optimized formulations from our previous published work [26] were prepared using a previously described thin-film hydration method [35] for MAP-loaded noisome and the cold method [36] for MAP -loaded ethosomes. Entrapment efficiency (EE%), particles size, zeta potential and polydispersity index (PDI) measurements of the prepared MAP-loaded niosomes and ethosomes were performed just as mentioned in our previous work [26]. All details about preparation and evaluation of MAP niosomes and ethosomes are mentioned in a supplementary file (Suppl.1).

### Preparation and characterization of Si-ZnO8 NPs gels containing MAP niosomes and ethosomes

Three different formulations were prepared using Carbopol gel 1% (1g), detailed preparation method is mentioned in (Suppl.1), and Si- ZnO8 NPs (1g) with either MAP ethosomes (2g) labeled as (Si-ZnO8-E), or MAP niosomes (2g) (labeled as (Si-ZnO8-N), or both (1g each) labeled as (Si-ZnO8-E-N) in addition to silica coated ZnO NPs gel (Si-ZnO8). Formulations were prepared through gentle physical mixing of all components and kept at refrigerator for further evaluation.

### Evaluation of the photoprotective effects of different nanoparticles preparations against UV induced damage in hairless mice

## Animals and experimental design

Female Swiss albino mice, 36 mice, (25–30 g) were obtained from the animal house of Faculty of veterinary medicine, Cairo University. They were divided into 6 groups, 6 mice each. The study design was approved by The Institutional Animal Care and Use Committee (IACUC), faculty of veterinary medicine, Cairo University. Approval No: (Vet CU 08072023679).

## Experimental Design

Group 1(G1): Normal control, no treatment, no UV exposure (NC).

Group 2 (G2): Photodamage model (UV) + vehicle.

Group 3 (G3): photodamage model (UV) + (Si-ZnO8) gel formulation.

Group 4 (G4): photodamage model (UV) + (Si-ZnO8-E) gel formulation.

Group 5 (G5): photodamage model (UV) + (Si-ZnO8-N) gel formulation.

Group 6 (G6): photodamage model (UV) + (Si-ZnO8-E-N) gel formulation.

## UV-irradiation Model

A UVA lamp (F 20W/T12/BL368; SYLVANIA; GERMANY) with a peak irradiance of 368 nm was used as UV source for UV irritation. Commercial hair removal cream was applied on the dorsal side on each mouse to remove hair 2 days before the experiments.

Animal cages were covered with a cellulose triacetate filter and kept under the UV lamp. Mice were pretreated with the tested formulations in a dose of 2 mg/cm^2^ one hour before each UV-exposure. The distance from the lamp to the backs of animals was 40 cm. Mice were allowed to move freely in the cage during exposure period. For each formulation, six mice were irradiated with UV daily for 5 days.

At the end of the experiment, mice were sacrificed 20 h after the last exposure to UV by cervical dislocation. Skin from all groups were harvested from the dorsal surface of the mouse and fixed in 10% formalin for histologic and immunohistochemistry studies [37].

## Histopathology and Immuno-histochemistry Study

Skin samples were taken from mice from all groups and were fixed in 10% formal saline for 24 h. After preparation, the obtained tissue sections were collected on glass slides and stained by hematoxylin & eosin stain [38].

Immunohistochemistry detection of NF-κB was done according to a previously reported method [39] using monoclonal antibodies to nuclear factor (NF)-κB p65 (Abcam, Cambridge, USA).

### Clinical assessment of the photoprotective effect of the prepared Si-ZnO8 NPs gels containing MAP niosomes and ethosomes

## Patients and Methods

### Recruitment of participants

Individuals with clinically diagnosed melasma and bilateral acquired facial hyperpigmentation were eligible to be enrolled in this study. Twenty patients (21–40 years old) were recruited from Dermatology outpatient clinic of El-demardash hospital, Egypt, during the period from March 2023 to September 2023. It was approved by the Research Ethical Committee, Faculty of Medicine, Ain Shams University. Study fulfilled all the ethical aspects essential in human research according to the Declaration of Helsinki Principles. All patients signed informed context after receiving full information about the treatment description, duration, and possible side effects. All patients were asked to stop any UV light therapy or sunbathing at least 30 days before starting this study. Also, they were asked to stop using any skin care products on their face during the study period.

Exclusion criteria: receiving any cosmetic treatments during the last 6 months before the study, women on any contraception, pregnant and lactating women, any skin disorders other than hyperpigmentation, as they may interfere with diagnosis or evaluation. Also, patients who are suspected to expose to excessive exposure to sun light and facial sun burn were excluded.

### Treatment and Regimen

Skin dermatological examination, after obtaining full history, for all patients was done using Antera 3D® Camera (Miravex, Dublin, Ireland). In our study, the patient facial skin was examined four times, at baseline (0 month), 1st month, 2nd month, and 3rd month. At each visit, the skin was examined and evaluated objectively for melanin level (Pigmentation), texture (Roughness index), and wrinkles depth (Indentation index).

Every patient was given two jars, the first one contained (Si-ZnO8) gel formulation and the second jar contained (Si-ZnO8-E-N) gel formulation and instructed to apply a fingertip amount, once daily at night, from one formulation on one side and the 2nd formulation on the other side to the hyperpigmented lesion on each side. Application was done for two months and stopped at the 3rd month. The first application was done at the end of the first visit under the supervision of a technician and patients were provided with verbal and written application instructions including cleansing step with water only. They were also instructed to report any changes that may appear in the form of itching, burning or erythema.

### Statistical Analysis

Statistical analysis of the collected data by one-way ANOVA, repeated measures test, was done using Graphpad Prism (GraphPad Software, San Diego, CA). All the data were described as the mean ± standard deviation (± SD). Comparison between Si-ZnO8 and Si-ZnO8-E-N was done using paired t-test after applying Bonferroni correction for multiple comparisons. Two-sided *p*-values less than 0.05 were considered statistically significant.

## Results and Discussion

### ZnO NPs Preparation and Coating with Silica

ZnO NPs were prepared using co-precipitation method. Zinc acetate was used as a source of soluble zinc ions and sodium hydroxide as the alkali. The resulted white precipitate of sodium acetate was removed from the obtained precipitate by washing. The obtained particles were dried at 120°C forming zinc alkoxide; the precursors of the ZnO NPs. Then cleavage of the alkoxide chains occurred by calcinations at 400°C to form ZnO NPs.

The introduction of the silica coating on ZnO NPs is more difficult than other metal nanoparticles because of the large difference in surface energy and the large surface area of ZnO NPs, therefore, they can be easily aggregated [40]. According to Stöber method for the introduction of the silica coating of ZnO NPs, the second strategy was followed [41]. This strategy suggests introduction of silica coat to previously prepared ZnO NPs which can consequently affect the shape, size, and dispersion of ZnO nanoparticles, and eventually their optical properties.

To investigate the effect of increasing TEOS concentration on the resulting silica coat, other factors were fixed. These factors included amount of ZnO NPs, alcoholic solution volume, time, and speed of stirring, in addition to ammonia concentration as ammonia acts as a catalyst that controls the deposited silica amount.

### Physico-chemical characterization of the prepared Si-ZnO NPs

#### UV–vis spectra (UV–Vis) and Photoluminescence (PL)

It is well known that some readable optical information is provided by the UV–visible spectra and Photoluminescence (PL) of nanoscale particles, including the intensity and position of absorption peaks. Also, some microstructural information concerning the nanocrystals can be dedicated from the UV–visible spectra, especially information about the estimation of suitable particle size [42].

**Fig. 1:**
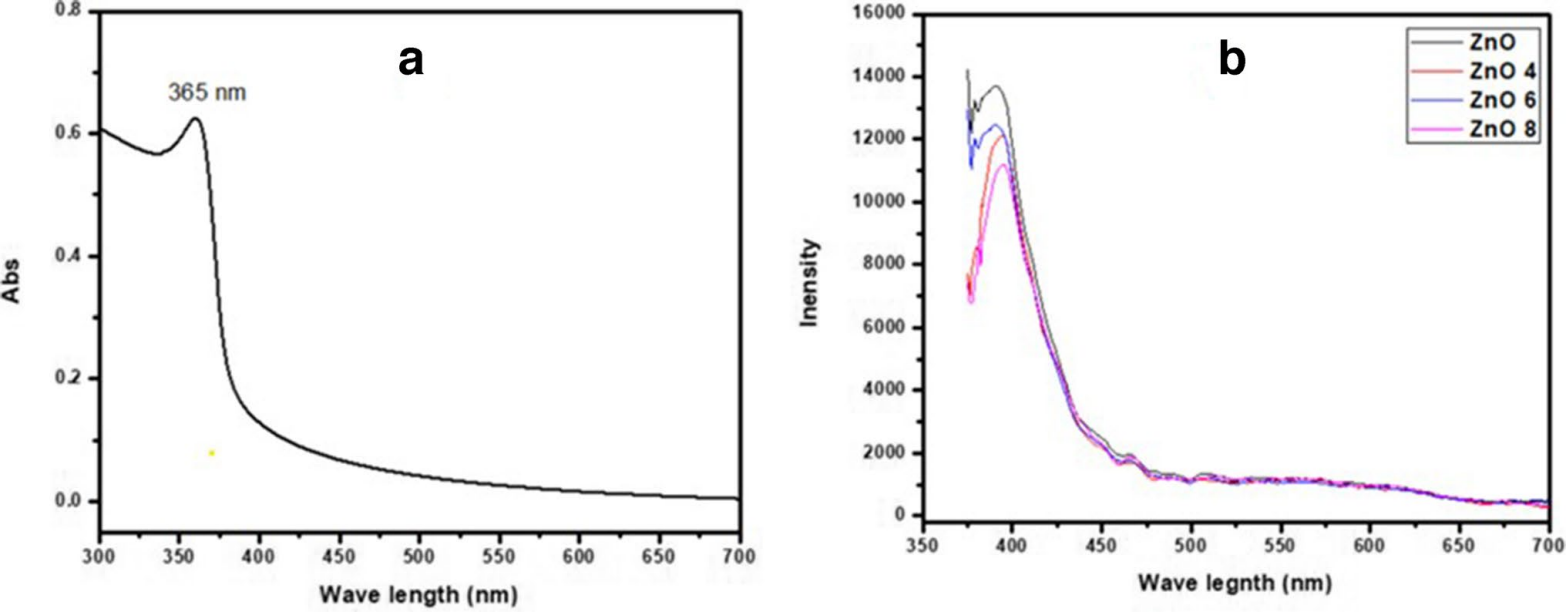
The optical absorption of ZnO NPs (**a**) and photoluminescence emission spectra of ZnO, Si-ZnO4, Si-ZnO6 and Si-ZnO8 NPs (**b**).

Figure 1 shows the absorption spectrum of pure ZnO NPs (a) and PL spectra of the prepared NPs after UV light excitation (b). All samples display the same emission behavior started at 375nm to 700 nm with slight shifting in the centered peak and the PL intensity which may reflect the structural changes of the nanoparticles due to coating with silica [42].

The PL intensity of all Si-ZnO NPs decreased significantly compared to non-coated particles. This is related to the silica coat which hinders the excited electrons from contributing in formation of free radicals after monochromatic light exposure. While, on the other side, the non-coated ZnO NPs are attacked easily after exposure to the strong light due to photoetching, the matter that makes them unstable [17, 43]. The greatest decrease in PL intensity was observed with the most thicker silica coat layer (Si-ZnO8) which can be used to make ZnO NPs as photostable particles with reduced formed reactive oxygen species (ROS) after exposure to UV light in agreement with our claim.

#### Crystal Structure: X- ray Diffraction

**Fig. 2:**
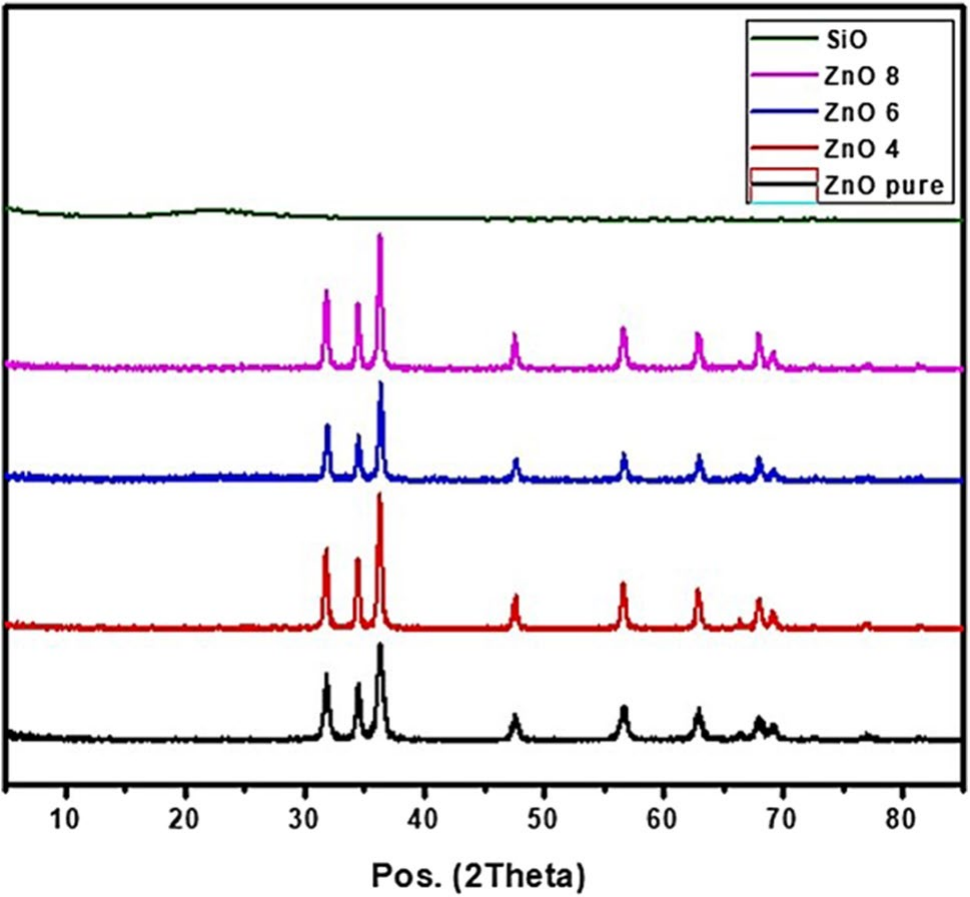
The X- ray diffractometers of the prepared samples.

The X-ray diffraction (XRD) pattern of the pure ZnO powder, pure SiO2, Si-ZnO4, Si-ZnO6 and Si-ZnO8 composites are shown in Fig. 2. The peaks at 31.7°, 34.4°, 36.25°, 47.55°, 56.6°, 62.87°, 66.4°, 67.97° and 69.1° in pure Zinc oxide are the characteristic of (100), (002), (101), (102), (110). (103), (200), (112) and (201) respectively (JCPDS card No. 01–089–0511) which implies the formation of crystalline ZnO NPs. Meanwhile, broad peak at 22° for pure silica assigned to amorphous structure of silica. All silica coated zinc oxide nanoparticles displayed the characteristic peaks of pure zinc oxide indicating their crystallin structure.

#### Morphology and Size Measurements

**Fig. 3:**
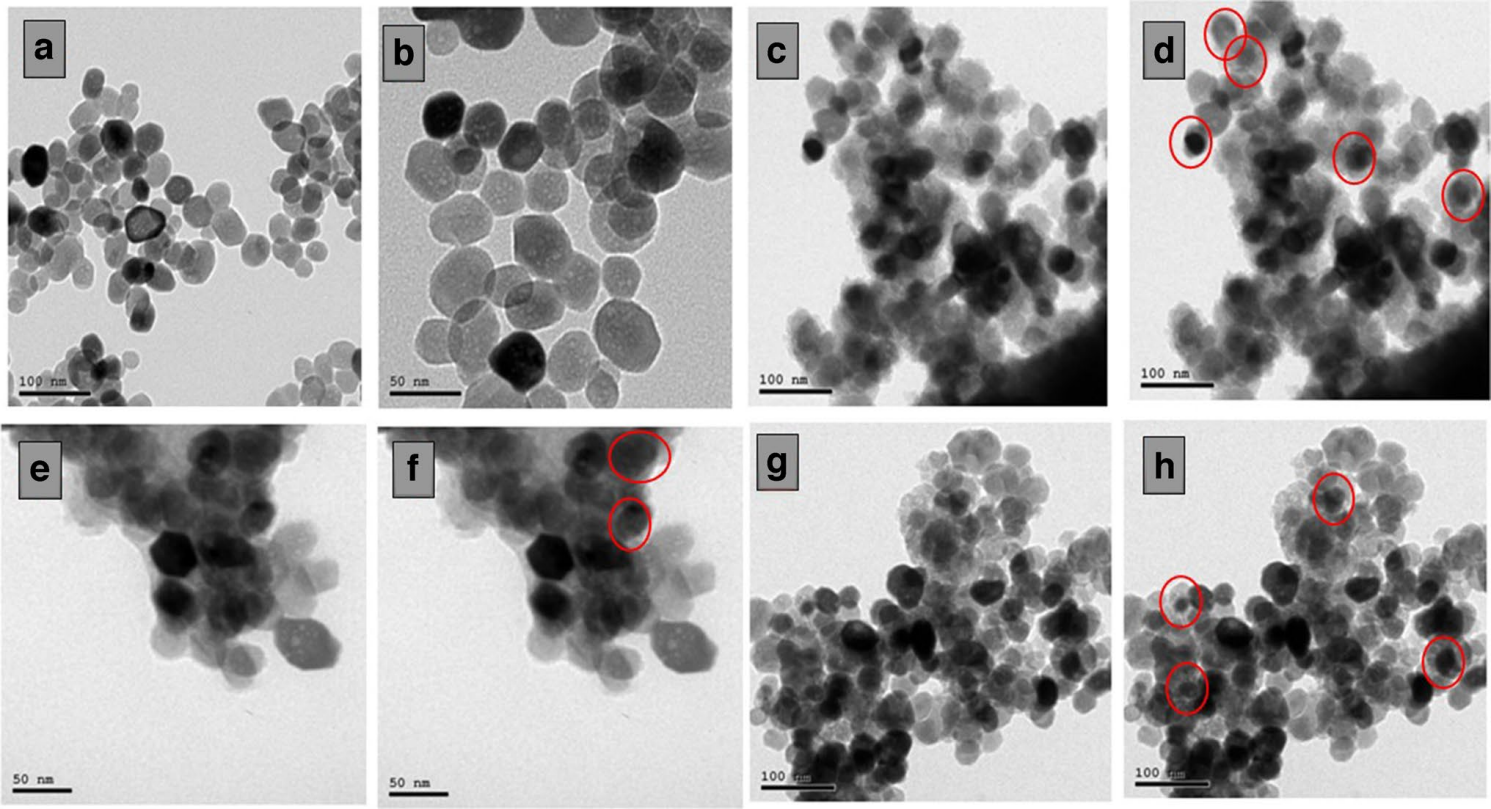
The TEM images of ZnO (**a**, **b**), Si-ZnO4 (**c**, **d**), Si-ZnO6 (**e**, **f**) and Si-ZnO8 (**g**, **h**).

Figure 3 revealed that the TEM images of ZnO NPs (a, b), Si-ZnO4 (c, d), Si-ZnO6 (e, f) and Si-ZnO8 (g, h) have monodispersed spheroidal shape with uniform distribution of the particles with average particle size ranging from 35 ± 8 to 64 ± 13 nm. For Si-ZnO nanoparticles, images c-h, the dark color in the inner space indicates the ZnO particle while the lighter one indicates the silica coat. This can be explained by the higher atomic number of Zinc (65) than that of Silica (28).

Particle size has been increased with increased amount of TEOS relative to ZnO amount, Table 1, as per the average of about 100 random particles measured for each sample. This means that increased TEOS amount resulted in increased the shell layer of silica around ZnO NPs.

**Table 1:** DLS and zeta potential values of Si- ZnO, Si-ZnO4, Si-ZnO6 and Si-ZnO8 NPs

Active material	TEM (nm)	DLS (nm)	Zeta potential (mV)
ZnO NPs	35 ± 8	165.8 ± 18.5	+ 25.4 ± 3.5
Si-ZnO4 NPs	49 ± 11	179.6 ± 20.2	-23.6 ± 6.8
Si-ZnO6 NPs	56 ± 9	183.7 ± 18.7	-32.8 ± 5.7
Si-ZnO8 NPs	64 ± 13	189.3 ± 19.4	-36.2 ± 6.8

#### Particle size (PZ) and zeta potential (ZP) measurements

**Fig. 4:**
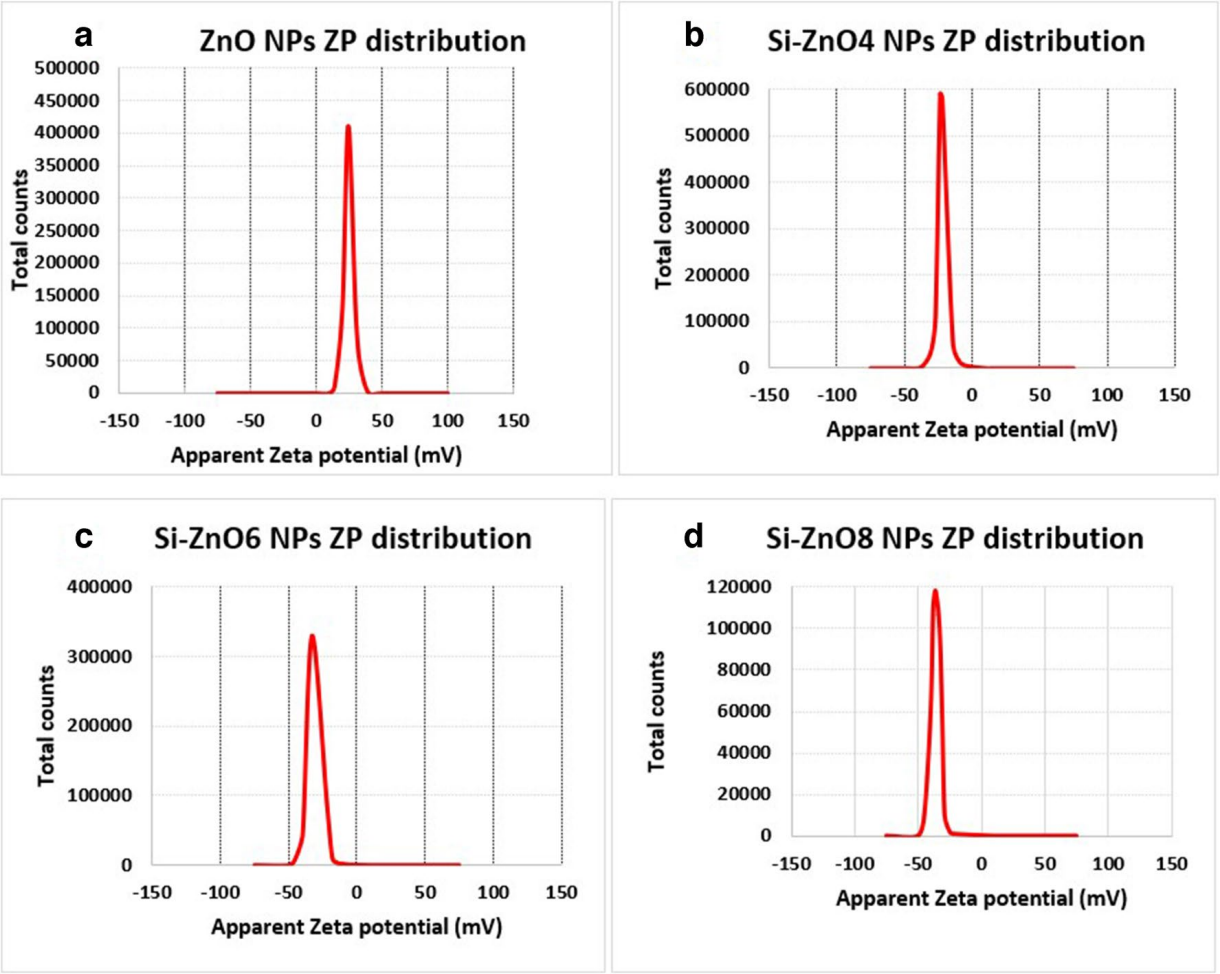
Zeta potentials (ZP) of ZnO (**a**), Si-ZnO4 (**b**), Si-ZnO6 (**c**) and Si-ZnO8 (**d**) NPs.

Zeta Potential analysis is a well-established technique that is used for determining the surface charge of nanoparticles in a colloidal solution, it typically ranges from + 100 mV to -100 mV [44]. The zeta potential values of the prepared nanoparticles are shown in Fig. 4. Un-coated ZnO NPs showed zeta potential of + 25.4 mV while after modification with silica the charge dropped to negative side in all samples as shown in Table 1. The negative zeta-potential value of silica-coated ZnO NPs is attributed to silica layer, having Si–OH groups on the surface resulting in regular distribution. This negatively charged surface layer participates strongly in decreasing cell toxicity caused by non- coated ZnO NPs as it was reported that the cell viability of anionic silica coated ZnO NPs increased relatively in comparison to cationic non-coated ZnO NPs as cationic NPs can interact strongly with the plasma membrane of the cell, which is negatively charged, and thereby induce high toxicological response [45, 46]. The gradual increase in zeta potential with increase in amount of silica coat suggests improved stability of the particle in presence of silica coat rather than un-coated ones.

**Fig. 5:**
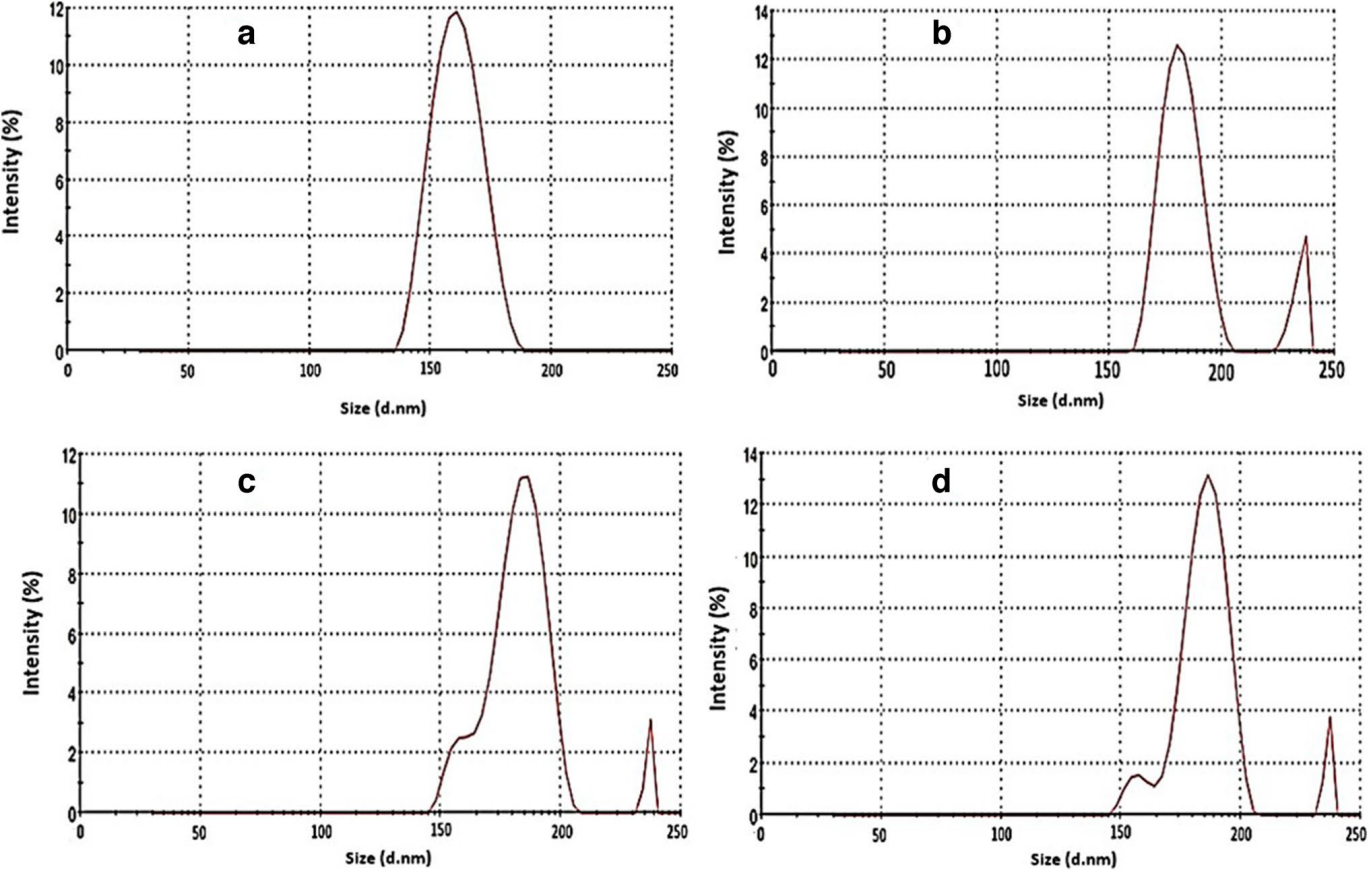
Dynamic light scattering (DLS) of ZnO (**a**), Si-ZnO4 (**b**), Si-ZnO6 (**c**) and Si-ZnO8 (**d**) NPs.

Dynamic light scattering (DLS) is a fast and relatively inexpensive tool to determine both the mean size and size distribution of a nanoparticle sample. The mean size determined by DLS is the equivalent spherical size. In this technique, each particle is assumed to be a perfect spherical particle [32]. All the prepared samples exhibited uniform size distribution, Fig. 5, with the hydrodynamic size of ZnO NPs increased from 165.8 ± 18.5 to 179.6 ± 20.2, 183.7 ± 18.7 and 189.3 ± 19.4 nm upon modification with silica ZnO4, ZnO6 and ZnO8 respectively, Table 1. This increase in size is due to an increase in TEOS molar ratio relative to ZnO resulting in thicker silica coat which agrees with our TEM images results and previously reported studies [17]. It can be observed here that the particle size measured through dynamic light scattering (DLS) is much larger than that measured through TEM for the same particles as the first technique depends on measuring the hydrodynamic diameter of hydrated nanoparticles in solution, which is usually larger than the size of dried nanoparticles observed by TEM [47].

#### Elemental analysis with energy dispersive X-ray spectroscopy (EDX)

**Fig. 6:**
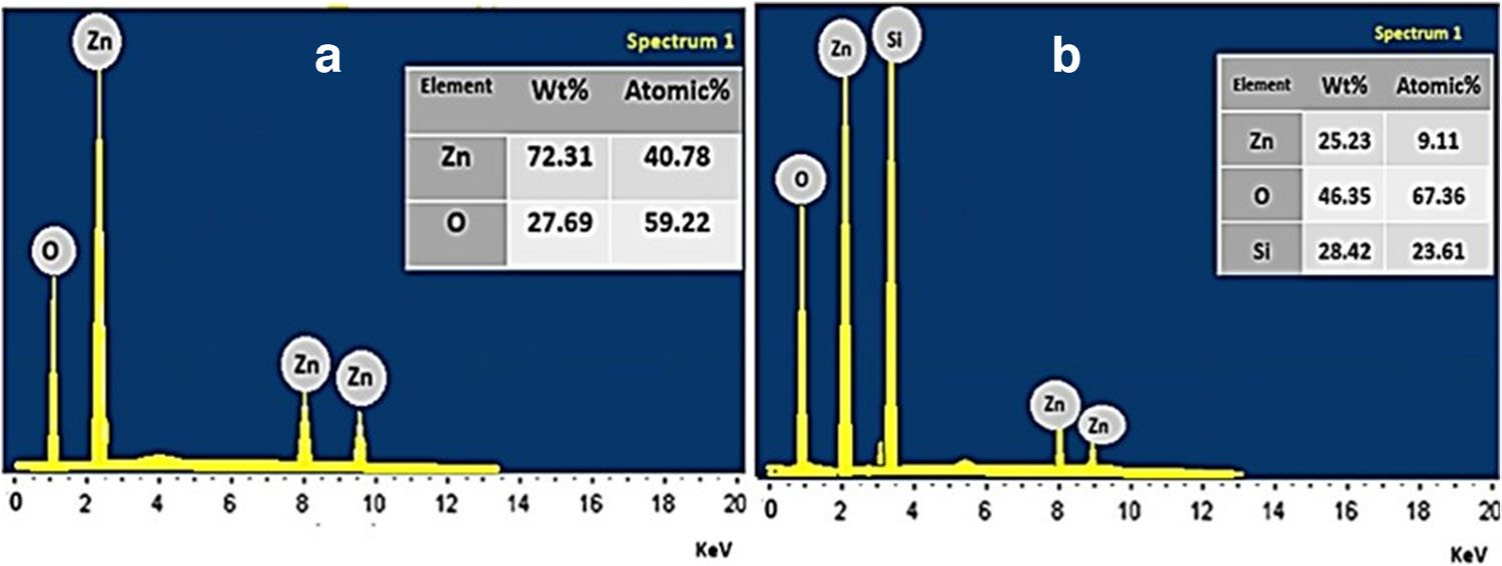
Elemental analysis with energy dispersive X-ray spectroscopy (EDX) of ZnO NPs (**a**) and Si-ZnO8 NPs (**b**).

Elemental analysis of ZnO NPs, Figure 6-a showed the main components of ZnO nanoparticles, oxygen and zinc, where that of Si-ZnO8 NPs showed additional peak of silicon (Si), Figure 6-b, conforming the coating of ZnO nanoparticles with silica.

#### Photocatalytic activity assessment and evaluation of reactive oxygen species (ROS) reduction

**Fig. 7:**
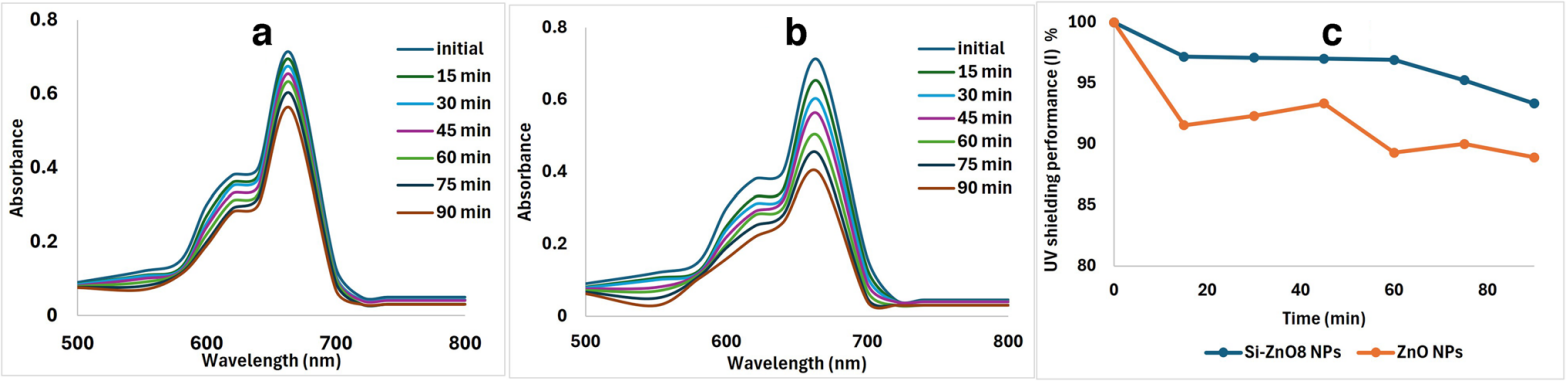
Photocatalytic activity assessment of ZnO NPs (**a**), Si-ZnO8 NPs (**b**) and their UV shielding performance (**c**).

As this study aimed to coat ZnO NPs with silica coat in order to reduce the potential generation of reactive oxygen species (ROS) after exposure to UV light and thus enhance their protective effect against UV irradiation. Evaluation of generation and utilization of ROS during exposure to UV irradiation was done through MB degradation assessment. Figure 7 shows the photocatalytic effect of un-coated ZnO NPs (a) and coated ones (b). Both formulations showed a decrease in MB peak intensity as the time of irradiation increases with significantly higher photocatalytic effect of ZnO NPs. This confirms that silica coat around ZnO NPs could decrease ROS generation upon exposure of ZnO NPs to UV irradiation and thus decrease their potential toxicity [16, 17]. As a result, the UV shielding performance was significantly higher for the silica coated ZnO nanoparticles, Fig. 7-c.

### Preparation and physico-chemical characterization of MAP niosomes and ethosomes

MAP ethosomes and niosomes were successfully prepared and evaluated. Their EE%, particle size, PDI and ZP were measured, Table 2, and they were found to have non-significant difference from the previously reported ones [26] indicating reproducibility of the followed method.

**Table 2:** Physico-chemical characterization of the prepared MAP niosomes and ethosomes

	EE%	PS (nm)	PDI	ZP
MAP niosomes	87.34 ± 3.85	146.16 ± 20.41	0.298 ± 0.091	-29.47 ± 3.11
MAP ethosomes	85.36 ± 3.52	151.74 ± 18.57	0.284 ± 0.086	-33.81 ± 2.94

### Evaluation of the photoprotective effects of different prepared nanoparticles preparations against UV induced damage in hairless mice

**Fig. 8:**
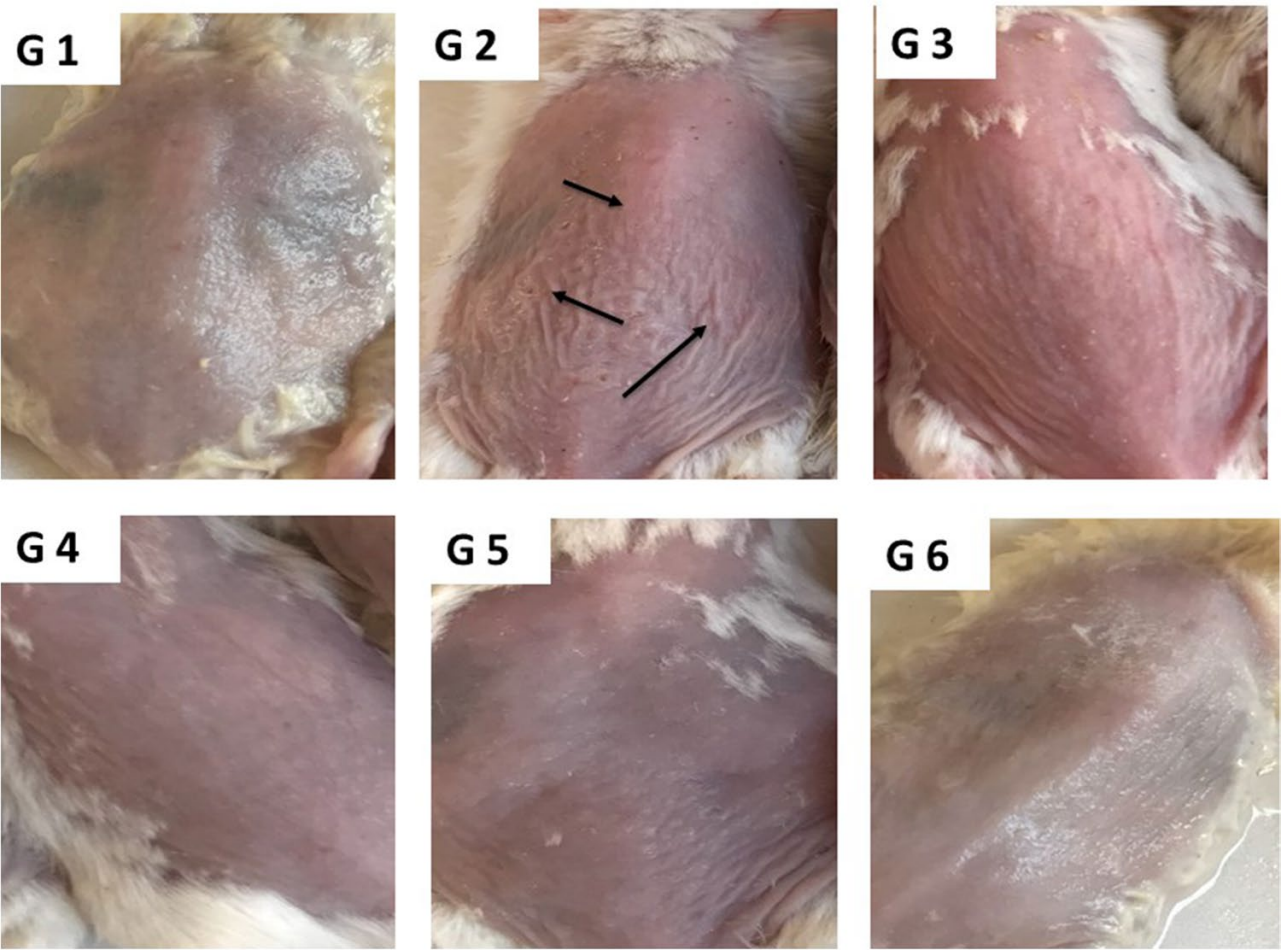
The typical appearance of hair-removed mouse skin after UV irradiation. G1: Normal control, G2: Photodamage model (UV), G3: photodamage model + control (ZnO8 NPs), G4: photodamage model + (Si-ZnO8-E), G5: photodamage model + (Si-ZnO8-N), G6: photodamage model + (Si-ZnO8-E-N).

Figure 8 shows the appearance of hair-removed mouse skin after UV irradiation with or without application of tested formulations. The figure reveals that UV irradiation, without application of any tested formulation, resulted in skin inflammation, erythema, roughness, and wrinkles (G2) compared to the control group (G1). On the other hand, pretreatments with different formulations resulted in low protection with (Si-ZnO8) gel formulation (G3), moderate protection with (Si-ZnO8-E) gel formulation (G4) and (Si-ZnO8-N) gel formulation (G5), while better protection was obtained with (Si-ZnO8-E-N) gel formulation (G6) that contains MAP ethosomes and niosomes in addition to Silica coated ZnO NPs.

#### Histopathological findings

**Fig. 9:**
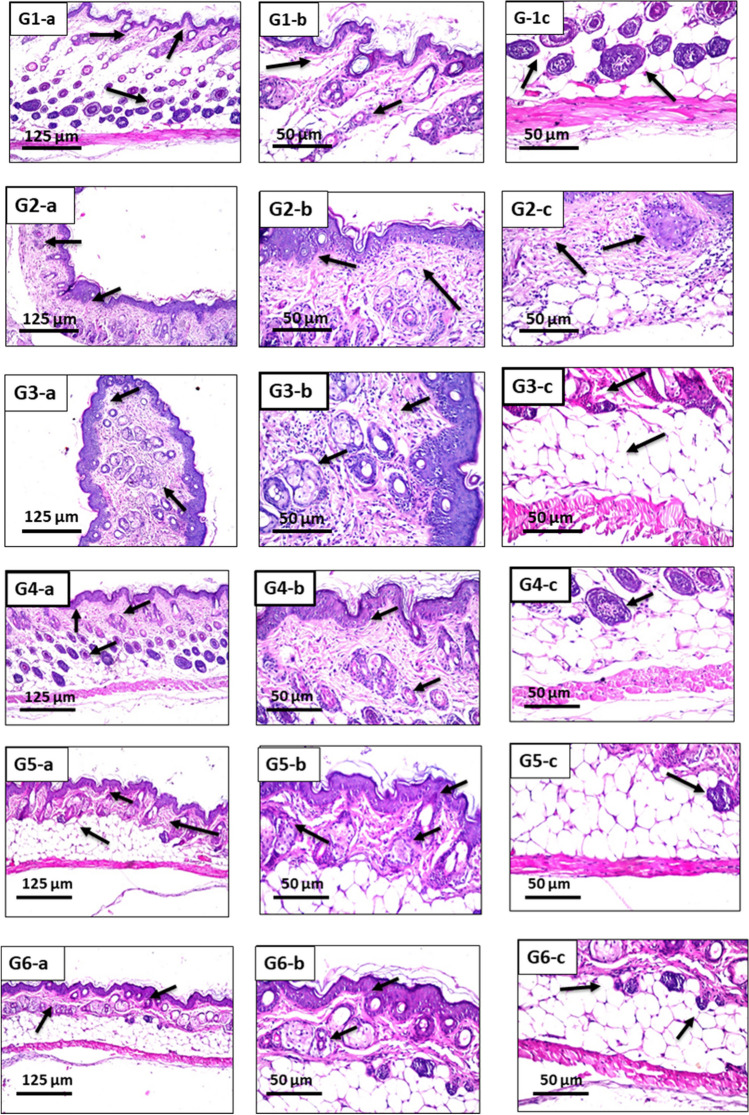
G1-a (× 16): skin of mice of G1, showing normal histological structure of the epidermis, dermis with hair follicle and sebaceous glands followed by subcutaneous tissue and musculature. G1-b (× 40): skin of mice of G1, showing the magnification of Fig. 1a to identify the normal histology of epidermal layer with underlying dermis containing hair follicles. G1-c (× 40): skin of mice of G1, showing the magnification of Fig. 1a to identify the subcutaneous tissue and musculature. G2-a (× 16): skin of mice of G2, showing focal acanthosis was detected in the epidermis while the underlying dermis showed inflammatory cells infiltration, hyperplasia in the sebaceous glands and necrosis in some of the hair follicles. G2-b (× 40): skin of mice of G2, showing the magnification of Fig. 2a to identify the focal acanthosis in the epidermis with hyperplasia of sebaceous glands and inflammatory cells infiltration in the dermis. G2-c (× 40): skin of mice of G2, showing the magnification of Fig. 2a to identify the necrosis of some hair follicles and inflammatory cells infiltration in the dermis and subcutaneous adipose tissue. G3-a (× 16): skin of mice of G3, showing acanthosis in the epidermis with inflammatory cells infiltration in the dermis. G3-b (× 40): skin of mice of G3, showing the magnification of Fig. 3a to identify the acanthosis in the epidermis and inflammatory cells infiltration in the dermis. G3-c (× 40): skin of mice of G3, showing the magnification of Fig. 3a to identify the normal histological structure of subcutaneous adipose tissue and muscles. G4-a (× 16): skin of mice of G4, showing mild acanthosis in the epidermis with few inflammatory cells infiltration in the dermis with intact underlying subcutaneous adipose tissue and muscles. G4-b (× 40): skin of mice of G4, showing the magnification of Fig. 4a to identify the mild acanthosis in the epidermis with few inflammatory cells infiltration in the dermis. G4-c (× 40): skin of mice of G4, showing the magnification of Fig. 4a to identify the normal histopathological structure of subcutaneous adipose tissue and muscles. G5-a (× 16): skin of mice of G5, showing mild acanthosis in the epidermis with few inflammatory cells infiltration in the dermis with intact underlying subcutaneous adipose tissue and muscles. G5-b (× 40): skin of mice of G5, showing the magnification of Fig. 5a to identify the mild acanthosis in the epidermis with few inflammatory cells infiltration in the dermis. G5-c (× 40): skin of mice of G5, showing the magnification of Fig. 5a to identify the normal histological structure of subcutaneous adipose tissue and muscles. G6-a (× 16): skin of mice of G6, showing normal histological structure of the epidermis, dermis, subcutaneous tissue and musculature. G6-b (× 40): skin of mice of G6, showing the magnification of Fig. 6a to identify the normal histology of epidermis and dermis. G6-c (× 40): skin of mice of G6, showing the magnification of Fig. 6a to identify the normal histology of subcutaneous tissue and musculature.

Figure 9 shows different histological effects after UV irradiation in different groups. For normal control group, G1, there was no histopathological alteration with normal histological structure of the epidermis and dermis. The hair follicle and sebaceous glands followed by subcutaneous tissue and musculature were recorded in normal state Fig. 9 (G1a, b, c)).

For G2 which wasn’t pretreated with any formulation while subjection to UV irradiation (Fig. 9 (G2a, b, c)), focal acanthosis was detected in the epidermis while the underlying dermis showed inflammatory cells infiltration, hyperplasia in the sebaceous glands and necrosis in some of the hair follicles. Pretreatment of mice with Si-ZnO8 gel formulation, G3, resulted in acanthosis detected in the epidermal layer while the underlying dermis showed inflammatory cells infiltration while there was no histopathological alteration in the subcutaneous adipose tissue and musculature (Fig. 9 (G3a, b, c)).

Combining MAP ethosomes and silica coated ZnO8 NPs, (Si-ZnO8-E) gel formulation, G4, resulted in mild acanthosis detected in the epidermal layer with few inflammatory cells infiltration in the dermis. There was no histopathological alteration in the subcutaneous adipose tissue and musculature (Fig. 9 (G4a, b, c)).

For mice pretreated with (Si-ZnO8-N) gel formulation, G5, UV irradiation caused the epidermis to show mild acanthosis associated with few inflammatory cell infiltrations in the dermis while the underlying subcutaneous tissue and muscles were intact (Fig. 9 (G5a, b, c)). For the final group, G6, in which the mice were pretreated with silica-coated ZnO8 NPs combined with both MAP ethosomes and niosomes, (Silica-ZnO8-E-N) gel formulation, there was no histopathological alteration in the epidermis, dermis, subcutaneous tissue and musculature (Fig. 9 (G6a, b, c)) and the histological structure was comparable to that of the control group that has no UV irradiation indicating the high ability of this formulation to protect the skin against the UV irradiation.

#### Immunohistochemical findings

**Fig. 10:**
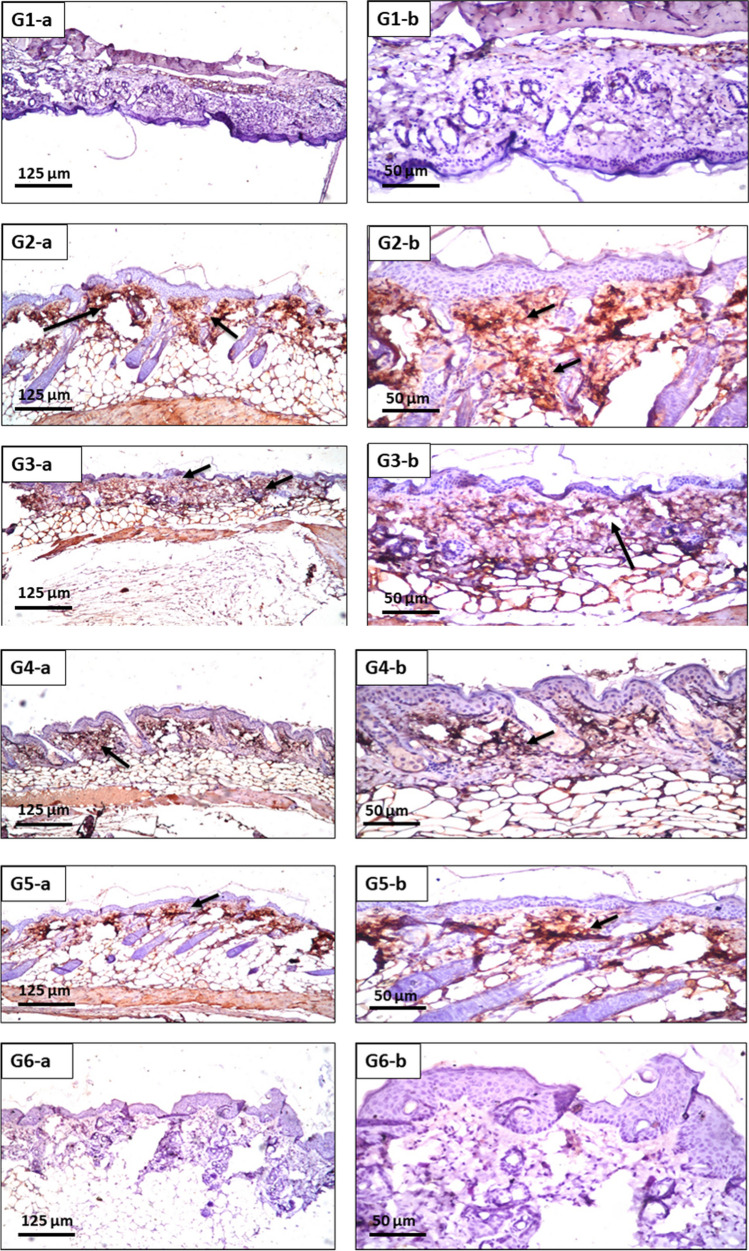
G1-a (× 16): skin of mice of G1, showing nil immunoexpression of NF-κB. G1-b (× 40): skin of mice of G1, showing the magnification of Fig. 1a. G2-a (× 16): skin of mice of G1, showing severe immunoexpression of NF-κB. G2-b (× 40): skin of mice of G1, showing the magnification of Fig. 2a. G3-a (× 16): skin of mice of G1, showing moderate immunoexpression of NF-κB. G3-b (× 40): skin of mice of G1, showing the magnification of Fig. 3a. G4-a (× 16): skin of mice of G1, showing mild immunoexpression of NF-κB. G4-b (× 40): skin of mice of G1, showing the magnification of Fig. 4a. G5-a (× 16): skin of mice of G1, showing mild immunoexpression of NF-κB. G5-b (× 40): skin of mice of G1, showing the magnification of Fig. 5a. G6-a (× 16): skin of mice of G1, showing nil immunoexpression of NF-κB. G6-b (× 40): skin of mice of G1, showing the magnification of Fig. 6a.

Figure 10 shows immunohistochemical findings for different groups. Un-pretreated group, G2, showed severe elevation in NF-κB immunoreactivity (Fig. 10 (G2a, b)) versus normal control group, G1, (Fig. 10 (G1a, b)). On the other side, all pretreated groups showed different degrees of NF-κB expression ranged from mild expression for G3, G4 and G5 to nil expression of NF-κB immunoreactivity for G6.

It was reported previously that chronic or acute exposure of skin to UV irradiation induces many harmful effects including impaired barrier function, inflammation, skin aging, and carcinogenesis [48–50]. These effects may be accompanied by erythema, edema, and immunosuppression [51, 52]. It has reported that, many of UV-induced skin harmful impacts are resulted from induction of many proinflammatory cytokines such as NF-κB [53].

Our results showed that all the applied formulation could enhance the histological and immunohistochemical skin protection against induced UV irradiation harmful effects. The superiority of this protection was obtained from the formulation containing combination of silica coated ZnO8 NPs, MAP ethosomes and MAP niosomes composites.

It is known that ZnO NPs are widely used in cosmetic products including sunscreens to filter the harmful ultraviolet sun light radiation (UVA (320–400 nm) and UVB (290–320 nm)) [54]. Ascorbic acid, represented as vesicular MAP nanoparticles, can protect the skin from the hazardous effects of ROS which leads to collagen breakdown, inflammation, skin aging and probably melanoma. It can protect skin from phototoxic damage, when applied topically [27]. Combination of both silica-coated ZnO8 NPs and MAP vesicular nanoparticles could protect skin against UV-induced histopathological alteration and NF-κB activation and affect the signaling pathways resulted from UV radiation in animal or human skin cells.

### Clinical assessment of the photoprotective effect of the prepared Si-ZnO8 NPs gels containing MAP niosomes and ethosomes

**Fig. 11:**
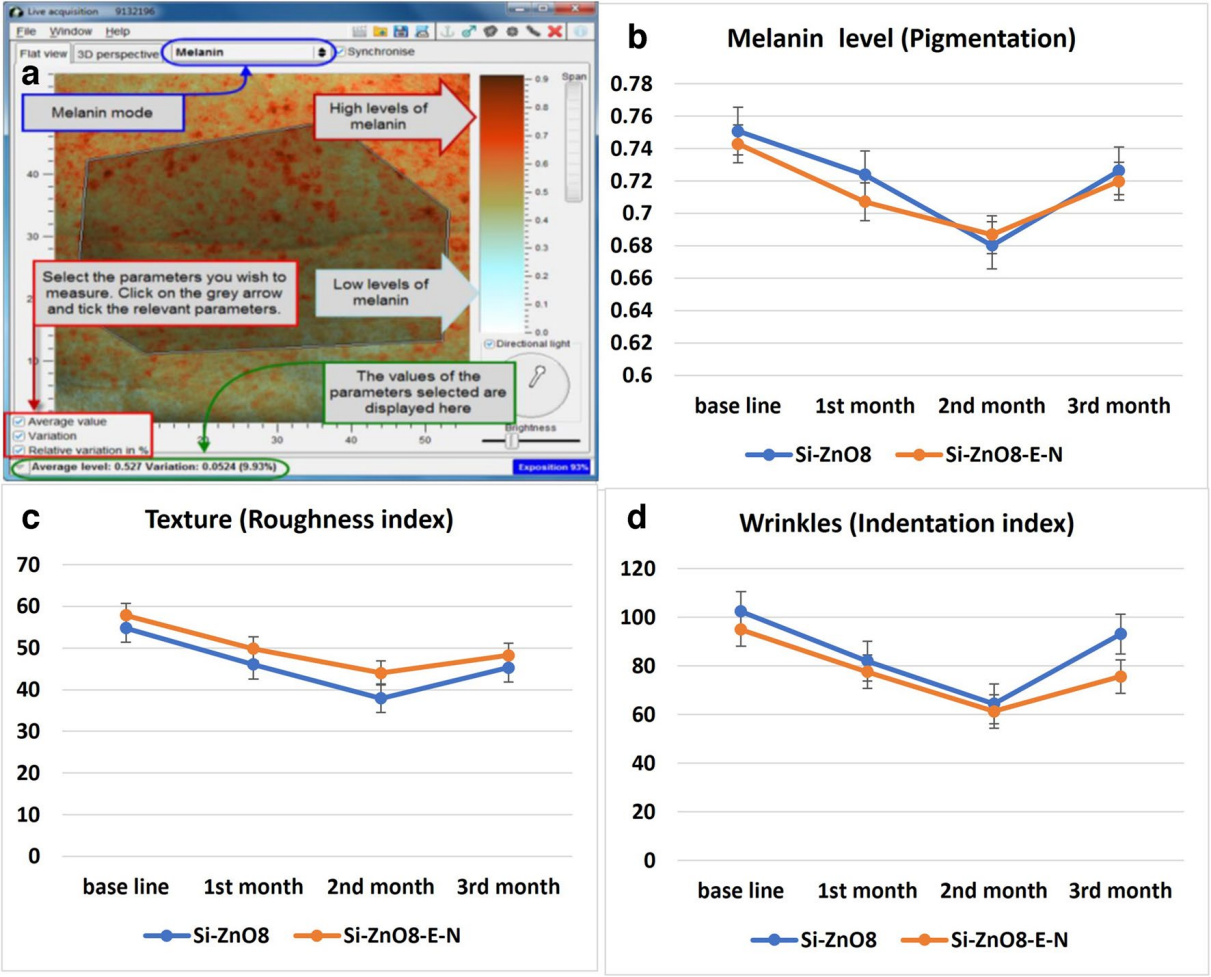
**a** Representative model of collecting data from Antera 3D® Camera, **b**: Effect of Si-ZnO8 and Si-ZnO8-E-N gel formulations on melanin levels, **c**: Effect of Si-ZnO8 and Si-ZnO8-E-N gel formulations on texture, **d**: Effect of Si-ZnO8 and Si-ZnO8-E-N gel formulations on wrinkles.

The age of patients enrolled to this study ranged from 21 to 40 years with mean age of 32.37 ± 5.13 year. In this study, Antera 3D® camera was used for quantitative analysis of melanin, texture, and wrinkles depth of facial skins. The Antera 3D® camera creates three-dimensional images with sophisticated mathematical algorithms and an optical technique. As a result, numerical objective data could be extracted from images, as shown in Fig. 11-a, which enables quantifying the treatments efficacy and monitoring different changes with time [26].

Photodamaged skin is usually characterized by many histological characters including amount of collagen reduction, collagen fibers fragmentation, elastotic degeneration of elastic fibers, dermal vessels dilatation, and epidermis atrophy and disorientation [55]. These characters are translated on the skin appearance as hyperpigmentation, wrinkles, and decreased skin elasticity. So, in our clinical study, melanin level (hyperpigmentation), wrinkles depth (Indentation index) and skin texture (Roughness index) were selected to evaluate the photoprotective effect of the tested formulations.

#### Melanin level evaluation

Results showed that both formulations, Si-ZnO8 gel and Si-ZnO8-E-N- gel have significantly changed the melanin levels with the time passed, each compared to its base line, Table 3 & Fig. 11-b. Melanin level decrease occurred after application for the 1st and 2nd month while it began to increase after stopping the treatment. T- test showed non-significant difference between the two formulations concerning their effect on melanin level, P value = 0.174.

**Table 3:** Statistical analysis of the photoprotective effect of Si-ZnO8 & Si-ZnO8-E-N gel formulations in a split face clinical study

Time	Melanin (Pigmentation)		Texture (Roughness index)		Wrinkles (Indentation index)	
	Si-ZnO8	Si-ZnO8-E-N	Si-ZnO8	Si-ZnO8-E-N	Si-ZnO8	Si-ZnO8-E-N
base line	0.75 ± 0.10	0.74 ± 0.10	54.79 ± 12.55	57.83 ± 10.35	102.45 ± 20.19	94.93 ± 18.68
1st month	0.72 ± 0.1	0.71 ± 0.10	46.03 ± 13.14	49.83 ± 10.26	82.03 ± 16.88	77.56 ± 16.65
2nd month	0.68 ± 0.09	0.69 ± 0.11	37.95 ± 8.64	43.99 ± 10.06	64.4215.45	61.35 ± 17.16
3rd month	0.73 ± 0.10	0.72 ± 0.10	45.28 ± 14.23	48.22 ± 12.43	93.12 ± 22.41	75.64.15.15
*P*- value/ significance	< 0.0001/ ***	< 0.0001/ ***	< 0.0001/ ***	< 0.0001/ ***	< 0.0001/ ***	< 0.0001/ ***
*F*- value	34.40	11.61	38.22	13.08	36.94	31.38
Si-ZnO8 # Si-ZnO8-E-N	0.174/ns		0.006/**		0,047/*	

^*^Significant

^**^Highly significant

^***^Extremely significant

#### Skin Texture (Roughness index)

Statistical analysis revealed significant effect (decrease) in the roughness index after treatment by both formulations, Table 3, Fig. 11-c, indicating enhancement in the skin texture. This enhancement decreased, through increase in the roughness index value, at the 3rd month with stopping the treatment. Significant difference was found between the effect of two formulations, *P* value = 0.006, with the superiority of Si-ZnO8-E-N gel formulation.

#### Wrinkles depth (Indentation index)

**Fig. 12:**
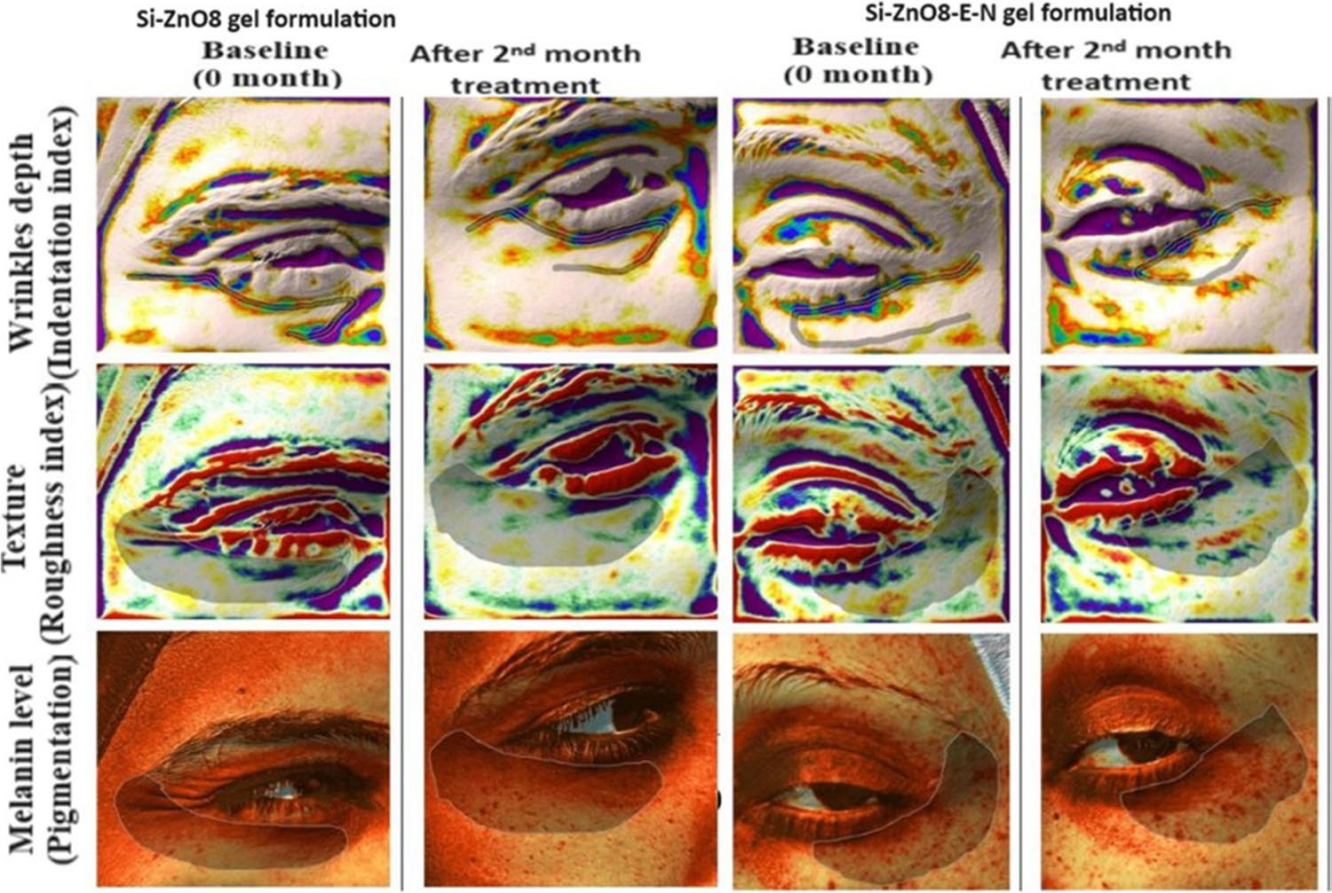
Representative examples of the involved patients’ photos taken by Antera 3D® camera for the melanin level, roughness index and wrinkles depth at different treatment times.

Significant effects of both formulations were found to decrease the wrinkles index after application for 2 months from the base line followed by elevation in the indentation index at the 3rd month, Table 3, Fig. 11-d, Si-ZnO8-E-N gel formulation was significantly better than Si-ZnO8 gel formulation, *P* value = 0,047, in decreasing the wrinkles depth. Figure 12 shows representative examples of the involved patients’ photos taken by Antera 3D® camera in our split-face study for the melanin level, roughness index and wrinkles depth at different treatment times using Si-ZnO8-E-N gel formulation.

It is worthy here to mention that no side effects such as redness or erythema were recorded by any conducted patient.

Silica coated ZnO NPs and vitamin C are widely used as photoprotective agents in many skin care products. Photocarcinogenesis, photoaging, and photosensitivity which are likely caused by exposure to the sun can be prevented by using suitable photoprotective agent [56].

Since it was reported that around 80% of skin aging, represented as increases in pigment heterogeneity and dermal elastosis, degradation of collagen, can be attributed to ultraviolet (UV) exposure. So, the best defence can be approached through introduction of an efficient photoprotective [57]. Our results showed that combination of silica coated ZnO NPs and vitamin C nanovesicles could represent an effective shield against UV- related photodamage. Vitamin C, being a predominant antioxidant, plays a vital role in the skin’s aqueous compartments due to its water solubility. Also, it helps replenish vitamin E, represent a cofactor in collagen synthesis, and help elastin accumulation reduction [58]. So, it can potentially improve the intrinsic defence systems of the skin. ZnO NPs are known to have UV blocking properties and ability to absorb and scatter UV rays. In addition, they were reported to have anti-oxidative property and inhibition of NF-κB pathway [19]. As a result, a combination of both, Si-ZnO8 NPs and MAP nanovesicles could enhance the photoprotective effect of each of them. More clinical studies with more investigation tools are required for accurate qualitative and quantitative effects of different combinations of both ZnO and Vit. C.

## Conclusion

Coating of ZnO NPs with silica could be used to make ZnO NPs as photostable particles with reduced formed reactive oxygen species (ROS) after exposure to UV light as indicated by decreased PL intensity of different ZnO NPs coated with different ratios of silica as well as higher UV shielding performance of Si-ZnO8 NPs over ZnO NPs. MAP ethosomes and niosomes were successfully prepared and evaluated with expected EE%, PDI and particle size. Application of different Si-ZnO NPs gel formulation as a protective against UV irradiation in mice resulted in no histopathological alteration in the subcutaneous adipose tissue and musculature with mild inflammatory cells infiltration compared to inflammatory cells infiltration, hyperplasia in the sebaceous glands and necrosis in some of the hair follicles in the un-protected skin. Addition of MAP nanovesicles to the Si-ZnO8 NPs gel formulation resulted in enhancement of the histological features of the skin after UV irradiation especially when ethosomes and niosomes were applied together. The obtained results were comparable to that of the control group that has no UV irradiation with nil expression of NF-κB immunoreactivity indicating the high ability of this formulation to protect the skin against the UV irradiation. Split-face study on 20 patients after application of Si-ZnO8 and Si-ZnO8-E-N gel formulations, one on each side, for two successive months indicated the efficient protective action of both formulations against photodamage effects as melanin level, roughness, and wrinkles without reported side effects with significant superiority for formulation containing both Si-ZnO NPs and MAP nanovesicles. As a result, this formulation could be a promising safe efficient skin care products component.

## Credit author statement

All authors have equally participated in the conception and design of the study, data acquisition, data analysis, and data interpretation. They have actively participated in drafting the article and revising it precisely for important intellectual content and have approved the final version to be submitted.

### Supplementary Information

Supplementary Material 1.
