# Supplementary material for: Duo photoprotective effect via silica-coated zinc oxide nanoparticles and Vitamin C nanovesicles composites
Source: Pharm Res. 2024 Jul 12;41(7):1475–91. doi: 10.1007/s11095-024-03733-y (PMC11263436; doi:10.1007/s11095-024-03733-y)
Supplement: Supplementary file 1 — Supplementary Material 1. [file 11095_2024_3733_MOESM1_ESM.docx]

**Supplementary documents**

**Pharmaceutical Research journal**

**Duo photoprotective effect via silica-coated zinc oxide nanoparticles and Vitamin C nanovesicles composites**

**Soha M. Kandil^1^, Heba M. Diab^2^, Amal M. Mahfoz^3^, Ahmed Elhawatky^4^, and Ebtisam M. Abdou^5^.**

**^1^Department of Pharmaceutics and industrial pharmacy, faculty of pharmacy, Modern University of Technology and Information (MTI), Cairo, Egypt.**

**^2^Department of dermatology, venereology and andrology, faculty of medicine, Ain Shams University, Cairo, Egypt.**

**^3^Department of Pharmacology and Toxicology, Faculty of Pharmacy, Modern University of Technology and Information (MTI), Cairo, Egypt.**

**^4^Department of Dermatology, Venereology and Andrology, National Research Centre, Cairo, Egypt.**

**^5^Department of Pharmaceutics, Egyptian Drug Authority (EDA), former; National Organization of Drug Control and Research (NODCAR), Cairo, Egypt.**

- 1. **Preparation of Magnesium-ascorpyl phosphate (MAP) nanovesicles (niosomes and ethosomes)**

Magnesium ascorpyl phosphate (MAP) niosomes and ethosomes optimized formulations from our previous published work [1] were prepared.

- - 1. **Preparation of Magnesium ascorbyl phosphate - loaded niosomes**

MAP niosomes preparation was done using a previously described thin-film hydration method [2]. In brief, the determined amounts of surfactant (Span 60, molar ratio of 2) and cholesterol (molar ratio of 1) were dissolved in 10 mL chloroform which was evaporated under reduced pressure using rotary evaporator (Rotavap, Type R110, Buchi, Switzerland) at 150 rpm and a temperature of 60 ± 2 ºC until a thin film was formed. The film was hydrated with 10 mL buffer solution (pH 7.4) containing the determined MAP amount (one molar ratio) and rotated until a suspension is formed. The suspension was cooled at room temperature and then subjected to sonication using a probe sonicator (SonifierVR 250 Branson, USA) in an ice-bath for three intermitted intervals each one for 5 minutes. The prepared niosomal dispersion was kept in the refrigerator at 4 ºC for further evaluation.

- - 1. **Preparation of Magnesium ascorbyl phosphate - loaded ethosomes**

MAP -loaded ethosomes were prepared using the cold method [3] in which phosphatidylcholine (1g/100 mL) and MAP (0.333 g/100 mL) were dissolved in an ethanol-propylene glycol mixture (50:10) and stirred using a magnetic stirrer (Wisestir, Wisd Lab. Instruments, USA) until complete dissolution. The mixture was heated to 30 ºC in a water bath. Pre-heated double-distilled water (up to 100 mL) was slowly injected using a syringe pump with constant stirring at 700 rpm in a closed vessel for 5 min. The resulted dispersion was sonicated at 4 ºC using a probe sonicator (SonifierVR 250 Branson, USA) for three cycles; each is of 5 min with 5 min rest between the cycles. Formulation was kept at 4 ºC for further evaluation.

# Physico-chemical characterization of the prepared MAP niosomes and ethosomes

# Entrapment efficiency (EE%) measurements

# Entrapment efficiency (EE%) of the prepared MAP niosomes and ethosomes was determined through ultracentrifugation of 2 mL of each formulation using a cooling centrifuge (Model 8880, Centurion Scientific Ltd., W. Sussex, UK) at 4 ºC and 15,000 rpm for 2 hours. The residue was washed three times each time with one-millilitre phosphate- buffered saline (pH 7.4) then was disrupted using 5mL ethanol, filtered, diluted with phosphate buffer and measured spectrophotometrically (Shimadzu spectrophotometer (Model UV- 1601, Japan) at a wavelength of 251nm to determine the MAP content. EE% was determined using the following equations:

# EE% = MAP amount in the vesicles/ MAP amount used in formulation * 100.

# Particle size, polydispersity index (PDI) and Zeta potential measurement

# Mean particle size, polydispersity index (PDI) and zeta potential measurement of the prepared MAP-loaded niosomes and ethosomes were performed using dynamic light scattering (Zeta-sizer Nano ZS-90, Malvern Instruments, Worcestershire, UK) after proper dilution.

- - 1. **Transmission electron microscopy (TEM)**

Transmission electron microscope (TEM) (JEOL JEM1230, Tokyo, Japan) was used to undergo the morphological examination of the prepared MAP niosomes and ethosomes. One drop of each sample was placed on a carbon coated copper grid to leave a thin film which was negatively stained with 1% phosphotungstic acid (PTA). The grid was left to dry, and samples were scanned under the transmission electron microscope operating at an accelerating voltage of 80 kV.

Results showed MAP niosomes as spherical vesicles with almost uniform size where MAP ethosomes appeared as sphere-like vesicles with dark coat and lighter core structure, **Figure (Suppl. 1).**


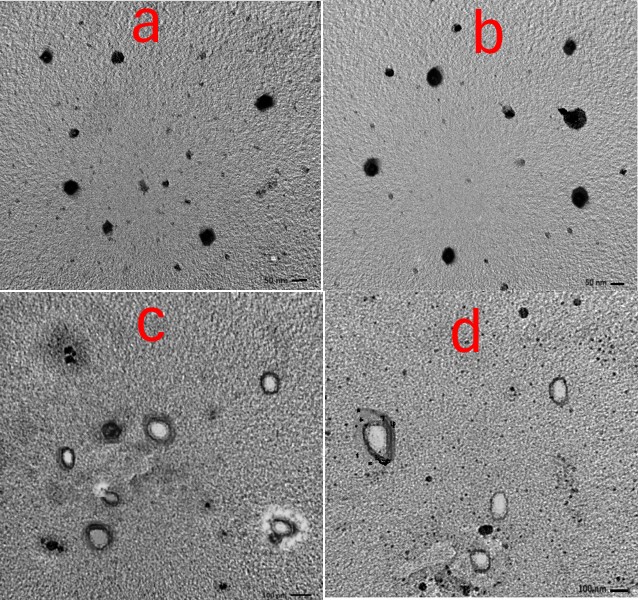


**Figure (Suppl. 1): TEM images of the prepared MAP niosomes (a, b) and MAP ethosomes (c, d).**

# Preparation and evaluation of Carbopol gel

# Carbopol gel (1% w/v) was prepared as described earlier [4]. Carbopol gel (1%) was prepared. One gm Carbopol 934 was soaked into 100 mL distilled water and kept 24 h to swell completely. Then, it was stirred using magnetic stirrer (Wisestir, Wisd Lab. Instruments, USA) at 500 rpm until homogeneity followed by the addition of few drops of triethanolamine to neutralize the pH and help formation of gel. The prepared gel was kept in the refrigerator overnight to get rid of any air bubbles. pH of the prepared gel was measured directly using a pH meter (Jenway 3540) and was found to be 7.1±0.3 which is in the physiological range.

**References:**

[1] Kandil SM, Soliman II, Diab HM, Bedair NI, Mahrous MH, Abdou EM. Magnesium ascorbyl phosphate vesicular carriers for topical delivery; preparation, in-vitro and ex-vivo evaluation, factorial optimization, and clinical assessment in melasma patients. Drug Deliv. 2022;29(1):534-547. doi: 10.1080/10717544.2022.2036872.

[2] Yeo LK, Chaw CS, Elkordy AA. The effects of hydration parameters and co-surfactants on methylene blue-loaded niosomes prepared by the thin film hydration method. Pharmaceuticals. 2019;12:46. DOI: 10.3390/ph12020046.

[3] Dina F, Eman MKY, Ghareb MS. Liposomal and ethosomal gels for the topical delivery of anthralin: preparation, comparative evaluation, and clinical assessment in psoriatic patients. Pharmaceutics. 2020;12: 446. DOI: 10.3390/pharmaceutics12050446.

[4] Aggarwal N, Goindi S. Dermatopharmacokinetic and pharmacodynamic evaluation of ethosomes of griseofulvin designed for dermal delivery. J Nano Res. 2013;15(10). https://doi.org/10.1007/s11051-013-1983-9
